# Bibliography

**Published:** 1898-10

**Authors:** 


### Bibliography.

A Treatise on Plateless Dentures. By C. A. Samsioe, practising dentist in Stockholm. With forty-eight illustrations. Translated from the Swedish by D. O. Bell. Published by the author, Stockholm.

It is not often that the pleasure is given English readers of having a translation of a book made in that language in a foreign country, and in the one in which it has been issued and under the supervision of the author. The novelty is, however, here presented, and upon the whole, excellently well done.

The work is upon "Plateless Dentures," a name given by Dr. Samsioe to express "artificial teeth secured in place, not by a plate covering the roof of the mouth, but in such a manner that the size of the prosthesis (?) will nearly correspond to the size of the defect. Thus under this heading come pivot teeth and crown- and bridge-work, whether stationary or removable."

The book is divided into ten chapters and one hundred and fifty-eight pages. The first part consists of a brief description of various crowns used in this country and elsewhere, exhibiting a very accurate knowledge of this kind of work.

Chapter three is devoted to treatment of living pulps, gangrenous pulps, pericementitis, etc.

The original character of the book is not shown until the sub-chapter on "How to make the Porcelain Facing and the Pivot (Impression)" is reached. That and the next sub-chapter of "How to cast the Backing from the Impression" cover practically all of Dr. Samsioe's method. In order that this may be understood the following quotations are given: "After the pivot and the crown have been fitted to the root and to each other we proceed to unite them and to take an impression of the root. For this purpose the operator should grasp the thickest end of the pivot with the pivot-tooth pliers and warm it over a spirit-lamp. A suitably large pellet of an impression composition, especially manufactured by me for this purpose, is then caused to adhere to the pivot by means of heat. This composition . . . is plastic, hardens quickly, adheres very firmly both to the porcelain tooth and to the metal, and does not change volume or form either in or after cooling. After the pivot and composition have again been slightly warmed the pivot is pushed into the root-canal as far as possible, whereby a perfectly true impression of the root is procured. . . .

"Then one of the small pins of the porcelain tooth, which are bent towards the cutting edge, is grasped with a pair of pliers and the tooth warmed over a spirit-lamp sufficiently for the operator to be able to press away the labial margin of the composition with a slight pressure of the tooth, and at the same time by melting it into the composition, secure the tooth in its right position in relation to the pivot and the other teeth in the row."

The author, after some advisory remarks, tells how to "cast the backing from the impression. The now fitted parts are invested in a little cast of plaster without admixture with sand or the like. Before the plaster has entirely hardened it is cut into a parallelopiped or cube. Besides this cutting, enough of the upper surface of the plaster is removed to leave the crown free a couple of millimetres below the cutting edge, so that it may afterwards be easily taken up out of the plaster. . . . If the composition is suitably warmed . . . it is possible successively to take up the crown, the pivot, and the composition without injuring the plaster or leaving any composition in the mould. The crown and the pivot are scraped clean and the small pins of the crown are bent and roughened as in ordinary rubber-work, but so that they neither collide with the pivot or with the plaster, nor come so high that they can stand in the way in the later articulation.

"When the crown and pivot are again in their right place, both the platina-iridium or alloy pivot and the pins are wet with a fine brush dipped in the soldering acid, care being taken that no acid touches the porcelain tooth or plaster.

"Over the opening of this depression a couple of pieces of the alloy made by me for this purpose are laid. This metal, which consists of tin, silver, gold, and platinum, in suitable proportions to make it melt readily and solder well, to make it tough, hard, durable, and capable of resisting the acids of the mouth, is to be had at the dental depots under the name of 'Samsioe's Metal for Plateless Dentures.' The piece of work is placed on a coarse wire net over a spirit-lamp, or a Bunsen or Fletcher burner. In about ten minutes, according to the size of the plaster ball and the strength of the flame, the metal begins to melt. With a large and strong pair of tweezers the piece of work is then lifted from the net, is knocked carefully (so that the tooth will not jump out) against something hard, so that the metal is forced down into the cavity of the plaster and surrounds the platinum pins. To make still more sure, the metal is pressed with a little piece of punk down into the cavity, and at the same time, just before the moment of hardening, the superfluous metal is wiped away, or it is forced up towards the cutting edge in order to form a chewing cusp. . . . If it is found that too little metal has been used, a little piece may be added to the melted mass, whose heat is frequently great enough to melt the added metal." The plate is then finished up.

The author gives many photographic illustrations of actual cases, not only of one tooth but a series of teeth inserted upon this method, which give a clearer idea of the work than words alone can convey.

While this character of work seems to be simple and readily adapted, it partakes of the objectionable feature of bridge-work, in that it is permanent, and, as represented, furnishes places for the lodgement of organic matter with the disagreeable results made familiar with that kind of work.

The book is well printed upon good paper, and, with the exception of a few typographical errors, it is not only a credit to the printers art in Stockholm, but a remarkable performance when all the circumstances of its production are considered.

